# First person – Daisuke Takao

**DOI:** 10.1242/bio.047670

**Published:** 2019-09-15

## Abstract

First Person is a series of interviews with the first authors of a selection of papers published in Biology Open, helping early-career researchers promote themselves alongside their papers. Daisuke Takao is first author on ‘Feedback loops in the Plk4–STIL–HsSAS6 network coordinate site selection for procentriole formation’, published in BiO. Daisuke conducted the research described in this article while an Assistant Professor in Daiju Kitagawa's lab at Graduate School of Pharmaceutical Sciences, University of Tokyo, Japan. He is now at the Graduate School of Medicine, University of Tokyo, investigating cilia and centrosomes.


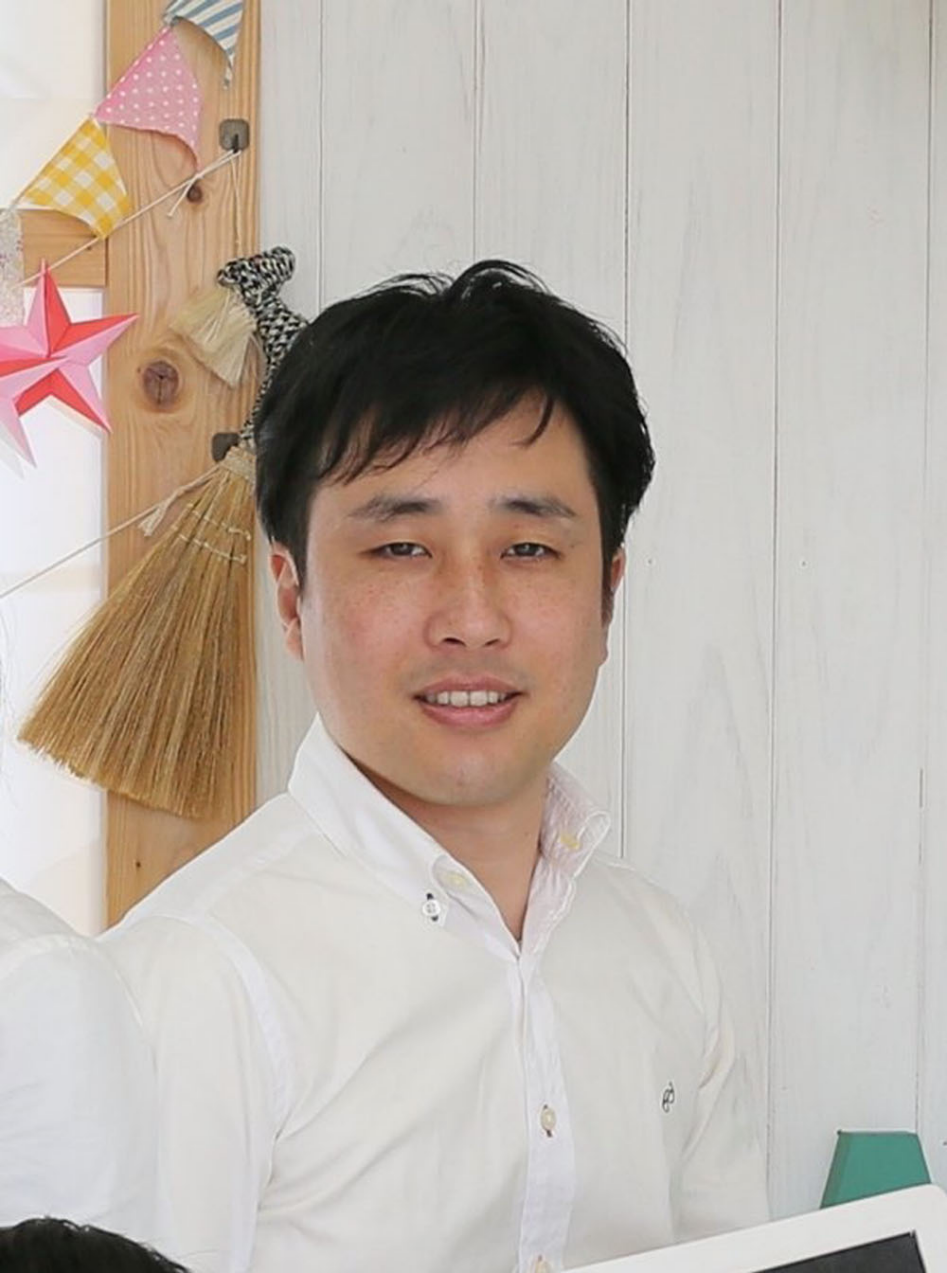


**Daisuke Takao**

**What is your scientific background and the general focus of your lab?**

I often face difficulties explaining my background in a word. I started my research career by working on biophysics of molecular motors. Most of my PhD period at the University of Tokyo was dedicated to development and application of optical microscopy combined with quantitative analyses and mathematical modeling to visualize molecular dynamics inside cilia. So I would say my background is biophysics. I also studied mouse development at the National Institute for Basic Biology, Japan. Then, in my post-doc period at the University of Michigan, I stepped into the cell biology field. Despite such an interdisciplinary background, my research has been consistently focused on cilia and centrosomes. It is exciting to think how molecular dynamics in such a small space organize complex cell systems.

**How would you explain the main findings of your paper to non-scientific family and friends?**

We successfully tracked the behavior of endogenous proteins functioning at centrosomes in live human cells, which was not as easy as it looked. A large body of work has presented an overview of such molecular behavior around centrosomes – such as changes in local protein levels along the cell cycle – in ‘fixed’ cells to provide snapshots of cellular processes. Our study, on the other hand, filled the gaps between those snapshots using live-cell imaging. Besides quantitative image analyses, we simulated the behavior of the molecules. Together, we demonstrated that our model including a complex network of molecular interactions can explain molecular mechanisms underlying biogenesis of centrosomal architecture. We established a way to directly visualize the dynamic behavior of endogenous proteins at centrosomes in live human cells with theoretical implications. I'm convinced this approach will be a new standard in the field.

“We established a way to directly visualize the dynamic behavior of endogenous proteins…”

**What are the potential implications of these results for your field of research?**

One of the most important messages from this study is in the approach. Once again I emphasize that we established a method to quantitatively track endogenous proteins at centrosomes in live human cells. This method includes techniques like CRISPR-Cas9 genome editing, spinning disc confocal microscopy and quantitative image analyses with original algorithms. As I mentioned, this study filled the gaps and connected the pieces of previous ‘snapshot’ information. So, someone might say such results are just within the scope of previous results and not surprising. I would say, ‘OK, hold on. You can say so only after actually looking at these results’. Even though there will not always be unexpected findings in the gaps of snapshots, such work is a significant part of the stream of science. I believe this work has advanced the field by proving we actually can look at what's going on at centrosomes in live cells.

**What, in your opinion, are some of the greatest achievements in your field and how has this influenced your research?**

Super-resolution microscopy has definitely changed cell biology, and of course the cilia and centrosome fields are no exception. The core architecture of cilia and centrosomes are as small as 200 nm in diameter, making them the best targets to test the limit of optical microscopy. Actually, a number of studies using super-resolution microscopy have gained our insight into the nanoscopic structures of cilia and centrosomes. Such achievements have been driving the field and of course I'm one to visualize the nanoscopic world with passion. I applied STED super-resolution microscopy in my recent work to visualize spatial patterning of molecules at centrosomes, which also motivated this work. Hopefully our achievements will encourage other scientists in the field as well.

**Figure:**
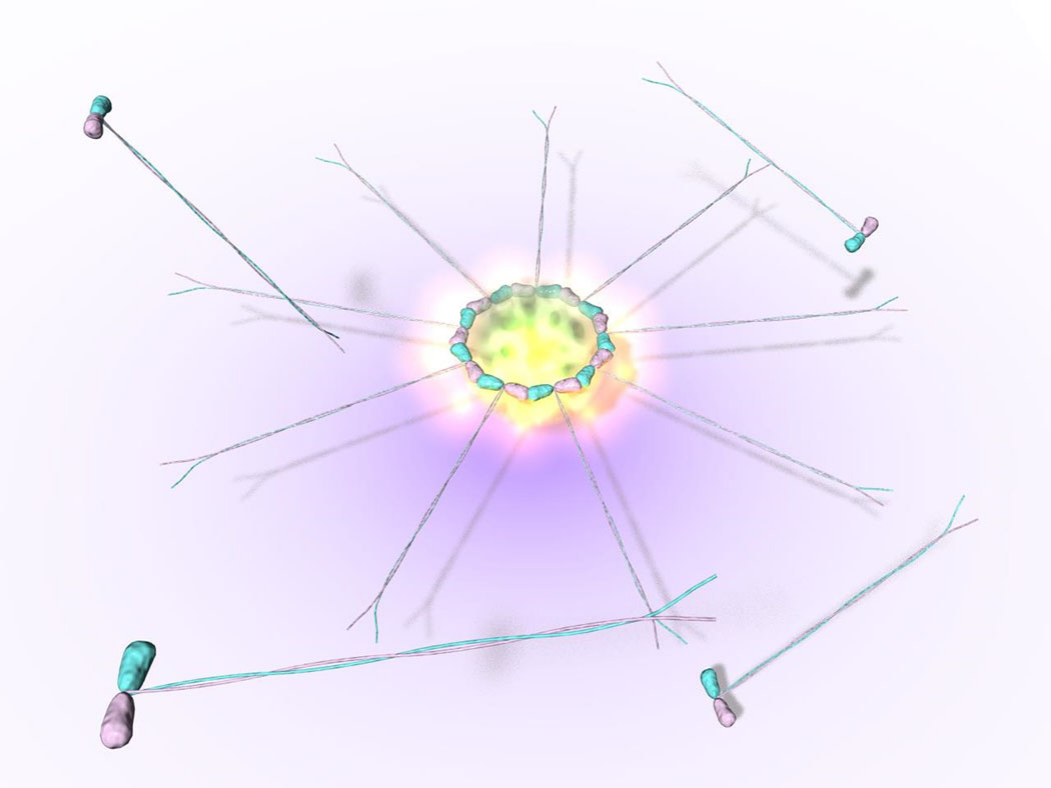
**Schematic of dynamic assembly of core centrosomal architecture.**

**What changes do you think could improve the professional lives of early-career scientists?**

Among a bunch of issues to discuss, I would pick the issue of excessive competition. Needless to say, we are forced to participate in the ‘impact factor race’. Is this really good for science? There could be alternative frameworks for assessing early-career scientists, instead of just short-sighted publication records. I know I'm talking about idealism, but I think many scientists are aware of this issue. I hope we can somehow find a better way to do good science with more creativity and less stress.

**What's next for you?**

Imaging is my life. I will keep imaging. Having more than 15 years as a microscopist, I want to keep looking at what exactly happens inside cells. It's so exciting to imagine how the behavior of molecules organizes the cellular system. Imaging approaches will surely help. I'll be focusing on cilia and centrosomes but also trying to expand my scope. Thinking about organelle communication will be an exciting option. How are molecular functions in cilia and centrosomes connected to those in other organelles? I do want to link those pieces in the context of the entire cell system.
